# A modified technique for radial artery access: how interventional radiologists can optimise the cardiologists’ technique to suite their procedures

**DOI:** 10.1186/s42155-024-00497-9

**Published:** 2025-01-02

**Authors:** Zaid Aldin, Josephine Weaver, Maha Khan, Tara Sadik, Viktor Manolas, Georgios Tsampoukas, Tariq Khatri, Marius Rebek, Ali Gharib, James Diss

**Affiliations:** 1 Radiology Department, The Princess Alexandra Hospital, Hamstel Road, Harlow, CM20 1QX UK; 2 General Surgery Department, Leeds General Infirmary, Great George Street, Leeds, LS1 3EX UK; 3 Urology Department, The Princess Alexandra Hospital, Hamstel Road, Harlow, CM20 1QX UK; 4 Urology Department, The Homerton University Hospital, Homerton Row, London, E9 6SR UK

**Keywords:** Prostate artery embolization, Vascular access, Radial access, Radial artery occlusion, Radial artery diameter

## Abstract

**Background:**

This is a single-centre prospective observational study examining radial access in 62 Prostatic Artery Embolisation (PAE) procedures. Evaluation of left radial artery diameter using high frequency ultrasound before and after administration of sublingual glycerl trinitrate (GTN). Pre-procedure questionnaires calculating symptom severity score compared with post-procedure.

**Results:**

Sublingual GTN resulted in a statistically significant increase in radial artery diameter (*p* < .00001). There was a statistically significant reduction in both average International Prostate Symptom Score (IPSS) and Quality of Life (QoL) from pre-procedure to post-PAE. The radial sheath was successfully inserted in 100% of cases. Crossover rate to femoral access was low (4%). Radial artery access had a low complication rate (2%). Radial artery variant anatomy was reasonably common (7%).

**Conclusions:**

Sublingual GTN significantly increase radial artery diameter. PAE from radial access is associated with a symptomatic improvement at 2-month follow-up.

## Objective

Single centre prospective observational study of a modified trans radial access (TRA) and distal radial access (DRA) technique for Prostatic Artery Embolisation (PAE) as an example of Interventional Radiology (IR) use of a Cardiology technique.

## Background

Radial artery access comprises typically of volar forearm radial artery access a few centimetres proximal to the radial styloid—also known as transradial access (TRA). RA has become the norm in many centres internationally for invasive coronary angiography and coronary intervention over femoral artery access (henceforth FA) due to a large body of high level evidence supporting its use. RA offers several advantages over FA, including a significantly lower risk of bleeding and vascular complications, quicker patient mobility, and a shorter recovery time, making same-day discharge more feasible. Furthermore, patients generally report greater comfort and satisfaction with RA.

However, Interventional Radiologists are largely behind the curve and continue to favour FA for vascular procedures such as visceral embolisation. There is an increasing use of RA amongst IRs but this is based on cardiologist’s techniques and experience. We describe why and how to optimise this access technique for the IR, using a single centre prospective observational study of PAE as an example procedure. To our knowledge, this is the first study to describe sublingual GTN use in RA. There has been a paucity of publications describing prostatic procedures from the radial artery [1–6]. As such, this study strengthens the existing evidence base.

## Methods

### Technique

#### Access

**Fig. 1:**
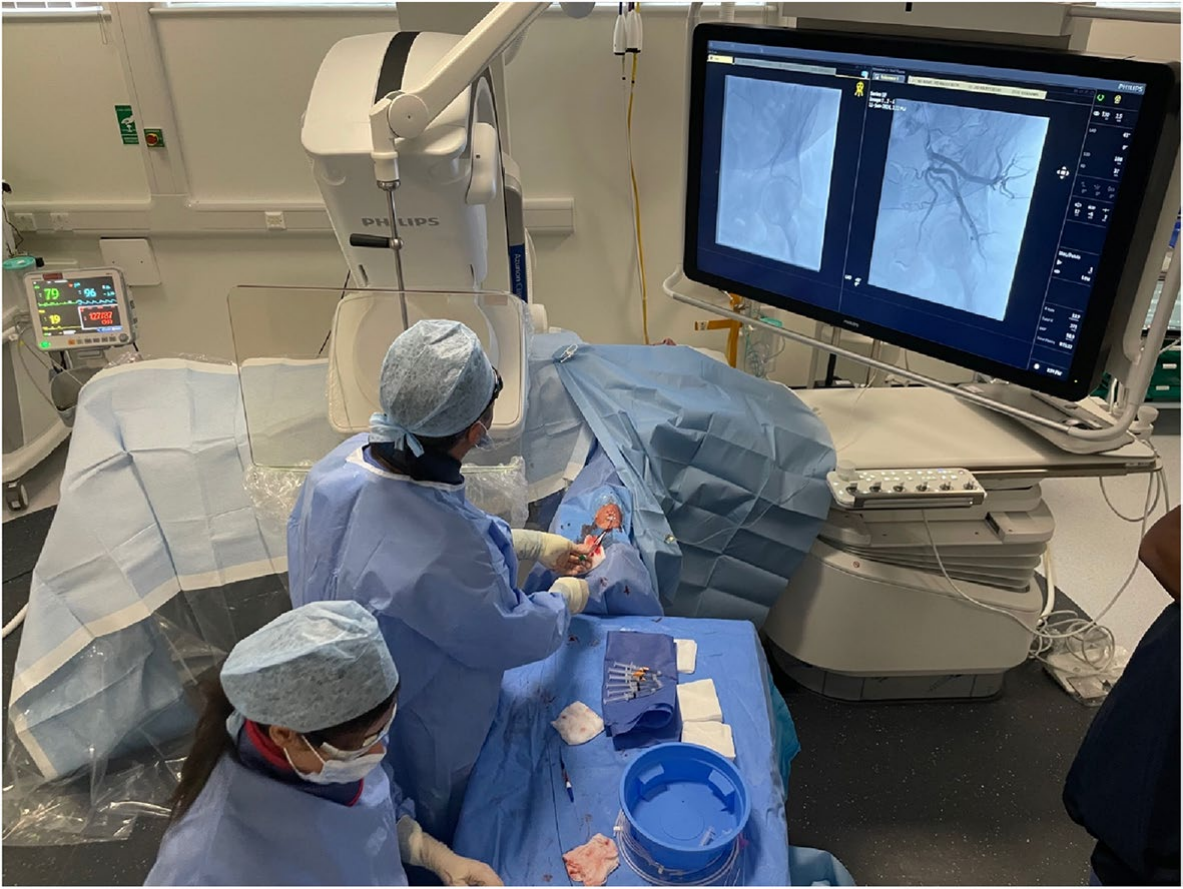
Room setup for PAE. The left arm is abducted on an arm board. The figure shows the positions of the operator, nurse, and technician, as well as the optimal placement of radiation protection and the angiographic machine (cone beam CT)

Position the patient’s left arm out on an appropriate arm board at a 45 (Fig. 1), with the hand in supination. Use a high frequency 10-15 MHz straight ultrasound probe (Acuson, Siemens, Munich, Germany) to assess the diameter of the distal radial artery, 2 cm proximal to the radial styloid (If the radial artery diameter is less than 2 mm, the patient is excluded from the study, and PAE is performed via the femoral approach). Evaluate the whole length of the radial artery in orthogonal views proximally to its origin at the brachial bifurcation. Determine if there is aberrant radial artery anatomy or a radial loop which may preclude RA. At the distal radial artery 2 cm proximal to the radial styloid, use spectral Doppler to evaluate the arterial waveform. Confirm triphasic flow, therefore significant subclavian artery stenosis/occlusion are excluded. Likewise, assess the whole length of the ulnar artery and check waveform at the distal ulnar artery. A radial loop is suspected on ultrasound when the artery bends sharply on itself, forming a loop or U-turn shape. Rather than following a straight or slightly curved path, the vessel folds back in the opposite direction before continuing its course. Colour Doppler typically shows turbulent or atypical flow patterns within the loop.

Measure non-invasive systemic blood pressure. Once establishing normotensive status, administer GTN 500 µg sublingually. After 5 min, re-evaluate the outer-to-outer radial artery diameter in the same position as previously. Prepare and drape the forearm as per local practice. Infiltrate 1-2mls 2% lidocaine subcutaneously under ultrasound guidance at the expected puncture site in the distal radial artery.

Perform ultrasound guided puncture of the distal radial artery using micro puncture access set (Cook Medical, Bloomington USA). Exchange for tapered radial 5Fr hydrophilic sheath (Terumo, Tokyo Japan). Connect the sheath to a continuous infusion pump of heparinised normal 0.9% saline at a rate of 12 ml per hour.

Conscious sedation via a peripheral venous cannula in the contralateral right antecubital fossa was given at the discretion of the operator according to patient preference. Where used, aliquots of 25 µg of fentanyl and/or 1 mg midazolam IV is preferred. Continuous monitoring of vital signs throughout the procedure is as standard.

#### Prostate artery embolisation

Using 135 cm or 155 cm bern tip 4Fr or 5F catheter (Merit Medical, Utah USA) and flexible hydrophilic guide wire (Terumo, Tokyo Japan), retrogradely traverse the radial, brachial, and subclavian arteries and access the descending thoracic aorta under fluoroscopic guidance. Administer initial loading dose of 3000 units heparin intra-arterially. Subsequent heparin is given 2 h into the procedure at a bolus dose of 1000 units per hour.

Selective cannulation of the prostatic artery is performed using a 175 cm microcatheter—TruSelect™ 2.0Fr (Boston Scientific, Marlborough USA) or Progreat Lambda™ 1.7Fr (Terumo, Tokyo Japan)—and 0.14 Fathom™ (Boston Scientific, Marlborough USA) guidewire. Administer 100 µg GTN intra-arterially to the prostatic artery via the microcatheter.

Additional to planar fluoroscopy, cone-beam CT imaging is at the operator discretion and technical availability.

Embolisation of the prostatic artery using 150–250 micron particles polyvinyl alcohol (PVA)—we use Contour™ (Boston Scientific, Marlborough USA)—in the distal part of the prostatic artery then larger particles 250–350 in the proximal part. Repeat on the contralateral prostate artery.

#### Patent haemostasis

Using TR BAND® Radial Compression Device (Terumo, Tokyo Japan) as per manufacturer's instructions. Apply the bladder of the device over the puncture site of the RA (ie. not the skin entry). Inflate the bladder with 15mls of air. We utilise continuous digital plethysmography technique for minimal pneumatic inflation with temporary manual compression occlusion of the ipsilateral ulnar artery. Sequentially deflate 1-2 ml air until patent haemostasis is achieved, followed by 30 min of stable inflation in the IR recovery area. Subsequently deflate 2mls serially every 10 min, monitoring for visual evidence of bleeding and haematoma. Should evidence of bleeding or haematoma be identified, 1-2mls air can be re-inflated as required. Patients are discharged home after a total haemostasis time of 1–2 h, once the TR band is completely deflated.

All patients received a follow-up phone call the day after the procedure to assess their recovery and address any concerns. They were provided with a direct contact number for the Interventional Radiology department for urgent issues during the day and were advised to attend the emergency department if needed at night. All patients were seen for a face-to-face, 30-min follow-up approximately 2 months after the procedure, during which IPSS scores and quality of life (QoL) measures were obtained, and any concerns were addressed. Radial pulses were clinically checked during follow-up to confirm radial artery patency. A weak or absent pulse was used as an initial screening tool for radial artery occlusion. Although the protocol included performing ultrasound if radial artery occlusion was suspected, this was not required, as no cases of occlusion were identified.

### Study design

This is a retrospective analysis of safety and efficacy of PAE via RA using our technique. All PAE procedures commenced in a single centre were included in the study over a 44-month period January 2020-August 2023 (*n* = 91). Two patients who had crossover to FA in a second procedure were still included for the purposes of evaluation of radial artery diameter. 28 primary FA procedures were excluded. A total of 62 procedures were suitable for analysis. All included patients for RA underwent ultrasound evaluation of the left forearm arteries. PAE via RA was performed by a single operator.

#### Primary end-point

RAD pre and post administration of GTN. All data was recorded on an Excel spreadsheet (Microsoft, California USA). Given the small sample size, we set our threshold p value at < 0.1.

#### Secondary endpoints

Puncture success, defined as successful insertion of the radial sheath. Reasons for conversion to FA were recorded.

International Prostate Symptom Score (IPSS), out of 35; and Quality of Life (QoL), out of 6; scores were measured pre and post-procedure.

Prostate volume (cc) was recorded pre and post-procedure.

Vascular access site complications and anomalous anatomy were recorded free text.

## Results

### Primary end-point

The mean RAD pre administration of GTN was 2.59 mm (standard deviation 0.42). The mean RAD post administration of GTN was 3.46 mm (standard deviation 0.56). Using one-tailed T-test, t-value = -9.76013. The *p*-value is < 0.00001. The result is significant at *p* < 0.05. Effect size is statistically significant using Glass's *delta* = (3.46—2.59) ⁄ 0.42 = 2.071429 (Tables 1 and 2).

**Table 1:** T-Test for radial artery diameter pre and post administration of sublingual GTN, assuming equal variance”

t-Test: Two-Sample Assuming Equal Variances			
Radial artery diameter (mm)	Pre	Post	
Mean	2.588709677	3.456451613	
Variance	0.172821259	0.317252776	
Observations	62	62	
Pooled Variance	0.245037017		
Hypothesized Mean Difference	0		
df	122		
t Stat	-9.760129595		
P(T < = t) one-tail	2.78E-17		**Significant**
t Critical one-tail	1.657439499		
P(T < = t) two-tail	5.56E-17		**Significant**
t Critical two-tail	1.979599878

**Table 2:** T-Test for radial artery diameter pre and post administration of sublingual GTN, assuming unequal variance

t-Test: Two-Sample Assuming Unequal Variances			
Radial artery diameter (mm)	Pre	Post	
Mean	2.588709677	3.456451613	
Variance	0.172821259	0.317252776	
Observations	62	62	
Hypothesized Mean Difference	0		
df	112		
t Stat	-9.760129595		
P(T < = t) one-tail	5.90E-17		**Significant**
t Critical one-tail	1.658572629		
P(T < = t) two-tail	1.18E-16		**Significant**
t Critical two-tail	1.981371815		

### Secondary endpoints

Puncture success was recorded as binary—either insertion of the sheath (1) or not (0). RA puncture was 100% successful.

Pre-procedure IPSS and QoL were recorded between one month prior up to the day of the procedure, most commonly at the time of pre-assessment clinic with the planning CT. Urinary bladder catheter IPSS and QoL scores were removed as incalculable. Post-procedure IPSS and QoL scores were evaluated at variable intervals post-procedure, usually at 2 months post-procedure (Table 3).

**Table 3:** Summary of paired 2-tailed t-Test results for primary and secondary outcome measures

-	–	Pre-treatment	Post-treatment
**IPSS**	**Mean**	20.4	7.5
	**Standard Deviation**	7.6	7.1
	**Observations**	38	35
	***p*-value**	< 0.01	
**QoL**	**Mean**	4.4	1.9
	**Standard Deviation**	1.4	1.9
	**Observations**	40	37
	***p*-value**	< 0.01	
**Prostate Volume (ml)**	**Mean**	126.6	97.6
	**Standard Deviation**	70.9	58.4
	**Observations**	54	23
	***p*-value**	0.076	
**RAD (mm)**	**Mean**	2.59	3.46
	**Standard Deviation**	0.42	0.56
	**Observations**	62	62
	***p*-value**	< 0.01	

The mean IPSS pre-procedure was 20.39. The mean IPSS post-procedure was 7.51. Using a one-tailed T-test, the t-value is 7.46434. The *p*-value is < 0.00001. The result is significant at *p* < 0.05.

The mean QoL pre-procedure was 4.375. The mean QoL post-procedure was 1.89. Using a one-tailed T-test, the t-value is 6.5537. The *p*-value is < 0.00001. The result is significant at *p* < 0.05.

There was a moderate loss-to-follow-up for post-procedure scores and group sizes for T-test were adjusted downwards accordingly.

Prostate volume (cc) was recorded pre and post-procedure. Typically this was measured on the pre-procedure planning CT pelvic angiogram and post-procedure on MRI prostate as part of follow-up. Alternatively, opportunistic measurements were taken from other abdominopelvic imaging performed for other reasons at an appropriate interval post-procedure (ie. > 2 months). Not all patients routinely underwent post-procedure imaging, again group sizes for T-test were adjusted downward. Given the contemporaneous nature of the study group, not all patients have yet reached a realistic post-procedure period to garner useful causal information about potential change in prostate volume (ie < 2 months).

The mean prostate volume pre-procedure was 127 cc, post-procedure was 98 cc. Using a one-tailed T-test, the t-value is 1.79933. The *p*-value is 0.037995. The result is significant at *p* < 0.05 (Table 3).

Three patients had high origin of the radial artery from the brachial artery in the arm.

One patient had a radial loop which could not be traversed. Anatomical variant rate was therefore 7%. One patient had a tortuous thoracic aorta which meant the prostatic arteries couldn’t be reached with the microcatheters and hence crossover to FA was required. Crossover rate was 4%.

Radial artery spasm was encountered occasionally despite the administration of sublingual GTN but were typically mild and did not impede the manipulation of the wire and catheter through the radial artery.

One patient had a moderate access site haematoma which resolved with conservative management and did not affect hand function, nor cause compartment syndrome. Overall complication rate of RA was 2%. We did not encounter any cases of radial artery occlusion.

## Discussion

Prostatic artery embolization (PAE) offers a safe and minimally invasive solution for addressing lower urinary tract symptoms (LUTS) associated with benign prostatic hyperplasia (BPH) [7]. Traditionally, PAE has been conducted through TFA. However, TRA provides multiple benefits, including enhanced safety profiles due to decreased complications at the access site. It contributes to patient satisfaction by enabling leg elevation during lengthy procedures, relieving lower back pain, and facilitating early post-procedure ambulation, which can assist in urination. Additionally, it offers potential cost savings for the department, including reduced reliance on closure devices used for transfemoral access, as well as shorter hospital stays and decreased postprocedural nursing care [1–6].

Visceral artery access may be advantageous via RA compared with FA as the angle of the mesenteric vessels ostia may be more favourable from the cranial approach. This may also be true of pelvic intervention in an ageing population with increased iliac vessel tortuosity.

Many vasculopathic patients requiring intervention have co-morbid conditions prohibiting groin access. Obesity, previous CFA endarterectomy healed surgical incisions, hernia repairs, and concurrent femoral vein cannulation can all contribute to “complicated groins” necessitating the use of alternative access such as RA.

RA confers a high technical success rate even in palpation guided puncture with low conversion rates to FA. Cardiologists rely upon the Barbeau test [8–13]- a modified Allen’s test to manually assess vessel patency. The Barbeau test evaluates the ulnar artery's ability to compensate for temporary occlusion of the targeted radial artery before performing TRA, with the aim of preventing ischemic hand complications. A pulse oximeter is placed on the patient's corresponding thumb to continuously monitor and record the waveform. The radial artery is compressed for two minutes, then released, and the pulse waveform is observed. Waveforms categorized as A-C suggest the patency of the ulnopalmar arch, indicating the safe feasibility of TRA. Conversely, waveform type D suggests inadequate arterial collateralization, thereby contraindicating TRA in such cases [6]. IR can improve success rate even further with the addition of ultrasound [14, 15]. Given our expertise in imaging, the need for performing the Barbeau test is obviated. Ultrasound quickly and effectively pre-empt radial associated issues:

- ➔ pre-determine if there is variant or aberrant anatomy of the radial artery [16]
- ➔ assess spectral Doppler to evaluate proximal stenoses/occlusion;
- ➔ assess the ulnar artery;
- ➔ measure the lumen size in order to approximate sheath Fr; and of course for guided puncture of the radial artery.

The Society of Interventional Radiology quality improvement standards on radial artery access [17] cite Barbeau waveform D as an absolute contraindication to RA. Our method obviates the need for Barbeau evaluation. In this study, the anatomical variant rate was 7%, which is similar to the rate reported in the published literature [15]. Specifically, three patients exhibited a high origin of the radial artery from the brachial artery in the arm, while one patient presented with a radial loop that could not be traversed. In patients with radial loops or suspected subclavian artery stenosis, identified through pre-procedural ultrasound, we opt for femoral access from the outset, using standard techniques. If a radial loop is discovered during the procedure and cannot be navigated, we discontinue the procedure, discharge the patient, and reschedule them for femoral access on a different day.

The technique proposed in this study involves administering 500 µg of sublingual glyceryl trinitrate (GTN) five minutes prior to puncture. The advantages demonstrated in this study include an increase in radial artery diameter, thus facilitating the radial artery puncture process, and a reduction in post-puncture radial artery spasm. Although occasional instances of radial artery spasm were encountered despite the administration of sublingual GTN, these occurrences were typically mild and did not hinder the manipulation of the wire and catheter through the radial artery. If necessary, an additional 100 µg of intra-arterial GTN were administered through the sheath or the catheter. It is noteworthy that in all 62 procedures included in this study, this approach was successfully implemented. We find this approach practical, easy, and time-efficient, enabling rapid puncture of the radial artery. We also administer the same dose of GTN sublingually to our PAE patients undergoing planning pelvic CT angiography so they are already familiar with its side effects prior to the procedure itself.

The most common side effect of sublingual GTN is headache, which is generally well tolerated. However, if the headache is troubling the patient, 1 g of intravenous paracetamol is administered to alleviate the discomfort. Less frequent side effects include dizziness and hypotension, though we have not observed these in our cohort. In the event that a patient develops these symptoms, intravenous normal saline may be administered at a rate appropriate to maintain stable blood pressure.

An alternative method, well-described in cardiology literature and frequently employed by interventional radiologists during radial punctures, entails applying a proprietary topical GTN cream (2%) or a blend of 2% GTN and anaesthetic cream (EMLA—Eutectic Mixture of Local Anaesthetics Cream) to the wrist an hour before the procedure [2, 5, 6]. However, we find this technique unnecessary and time-consuming. We advocate for the use of sublingual GTN as a more efficient option, enabling the commencement of the procedure in a shorter time.

Similarly, cardiology and radiology literature describe the use of local anaesthesia consisting of a mixture of 1 mL of 100 μg of GTN and 9 mL of 1% lidocaine delivered to the subcutaneous tissues around the left radial artery under ultrasound guidance [2, 5, 11]. In our experience, 2–3 ml of 1% lignocaine is sufficient for local anaesthesia.

Various 'cocktails' have been proposed to prevent radial artery spasm post-puncture [2, 5, 6, 12]. The most commonly used cocktail includes 2000 IU of heparin, 200 mg of GTN, and 2.5 mg of verapamil. However, this “cocktail” causes discomfort in the hand when injected. To reduce this discomfort, these medications are drawn up into a 20 mL syringe, and following access, blood is aspirated into the syringe to the full 20 mL volume via the sheath, then reinjected over 30 s. Despite the dilution and slow injection, many patients still experience discomfort in the hand, and verapamil's negative inotropic effect can induce hypotension, particularly at doses > 2.5 mg. We utilize smaller sheaths, 4F and 5F, compared to the standard 6F and 7F sheaths used by cardiologists, increasing the likelihood that cardiologists will encounter radial artery spasm, necessitating higher doses and frequencies of antispasmodic medications. In our experience, administering sublingual GTN just before starting the procedure significantly reduces radial artery spasm. Once the sheath is inserted, it is connected it to a continuous infusion pump of heparinized normal 0.9% saline at a rate of 60 ml per hour. Then, as the catheter and wire are manipulated to the aortic arch, 3000 IU of heparin is administered in the arch, followed by 1000 units of heparin every hour after two hours of procedure time. As the heparin is injected in the aortic arch and not in a small artery like the radial artery it will not cause any discomfort to the patient. Based on our experience, we have not encountered issues with arterial cannulation. In the event of a delay caused by significant arterial spasm, we recommend administering 2000 units of heparin directly through the sheath or the catheter, diluted with blood and injected slowly. This aims to minimize the risk of thrombosis or dissection. For patients with high risk for thrombo-embolism, we recommend adhering to general clinical and hospital guidelines to tailor anticoagulation therapy. In cases where heparin is contraindicated, femoral access, should be considered for PAE.

Recent IR studies have investigated the effectiveness of TRA and concluded that there are no significant differences in adverse events, radiation exposure, procedure duration, or clinical success rates compared to TFA [3, 4]. In a recent retrospective analysis, the procedural outcomes and adverse events of 998 patients undergoing PAE at a single center between April 2014 and August 2022 were examined [5]. Of these, 821 patients (82%) underwent TRA PAE, while 177 patients (18%) underwent TFA PAE. TRA PAE demonstrated comparable technical success to TFA PAE, with lower incidence of access site hemorrhagic complications and reduced radiation requirements. However, the interpretation of the findings regarding the favorable procedural time and radiation profile for TRA versus TFA is complicated by the learning curve and subsequent preference for TRA by the single interventional radiologist.

Another advantage of TR is operator ionizing radiation dose is most likely reduced when using TRA vs TFA given the increased distance from the primary beam [18]. As mentioned above. patient radiation dose and procedure time are largely unaffected by access in PAE [3, 4] and in cardiology cases [18–21].

While cardiologists typically favour the right radial artery for radial access (RA), we advocate for the use of the left radial artery. This approach eliminates the need to traverse the aortic arch during typical infradiaphragmatic interventions, thereby minimizing contact with major vessels and theoretically reducing the risk of stroke. It is important to acknowledge the rare complication of stroke; however, it is crucial to put this rare risk into perspective. Reported data often refers to asymptomatic strokes and includes cardiology procedures that involve crossing the arch in each case. The reported stroke rate in the published radiology literature about TRA PAE is 0.2 to 0.7% [5, 6].

Both pneumatic (for TRA) and elastic (for DRA) patent haemostasis devices are readily available and inexpensive [22]. “Do-it-yourself” alternatives and manual compression are also viable [23]. All options have a relatively short learning curve compared with closure devices typically used for antegrade or retrograde FA. Short duration and lowest required pressure for patent haemostasis is recommended to reduce the risk of RAO.

RAO is the biggest access related complication from RA, occurring 2–3% of the time [8, 24]. Pathophysiology of acute RAO results from one of, or a combination of: thrombosis, dissection, local hypercoaguable states, and/or compressive haemostasis (low flow). Due to significant collateral supply (especially in the case of DRA using the superficial arch) RAO is usually asymptomatic [25].

Peri-procedural heparin, either systemic or intra-arterial at a recommended dose of 5000 units and aspirin have been demonstrated to reduce RAO. We do not routinely commence aspirin pre-procedure for our PAE patients, but we do not discontinue it if it has been prescribed for another reason. We describe our heparin regime, 3000 IU of heparin administered in the aortic arch, followed by 1000 units of heparin every hour after two hours of procedure time, in combination with continuous infusion of heparinised saline throughout the procedure to reduce the risk of thrombosis around the sheath and subsequent RAO. This will also reduce the initial heparin bolus required and reduce bleeding risk [26]. Sheath size is an important consideration for RA, as sheath:artery ratio > 1 is associated with increased RAO risk. In fact, The Society of Interventional Radiology quality improvement standards on radial artery access cite “Radial artery anterior–posterior inner-to-inner wall diameter on ultrasound not compatible with the outer diameter of the introducer sheath” as an absolute contraindication to RA. We exclude any patient with radial artery diameter less than 2 mm. Multiple puncture attempts are associated with increased RAO rates and ultrasound guidance should reduce puncture attempts and as such reduce RAO risk further.

In PCI, RA is associated with reduced bleeding risk compared with FA. This may be in part due to the forearm offering only a small potential space around the puncture site wherein haematoma can collect unnoticed [27]. In cases of PCI, the patients are often receiving therapeutic anticoagulation and antiplatelet medication. In our elective PAE procedures, these can be temporarily withheld, in line with European best practice (CIRSE anticoagulation and antiplatelet protocols). As such, this should further reduce the bleeding risk associated with vascular access via RA. However, compartment syndrome is still a potential complication of RA [28], radial artery pseudoaneurysm [29, 30] and radial artery avulsion [31] have all been reported in RA.

RA is associated with early patient mobilisation, even over FA with successful deployment of closure devices. As a result, patients will need less nursing care. A survey published in 2020 revealed nurses tend to prefer radial over femoral access when caring for patients who have undergone interventional radiology procedures, independent of years of experience [32]. However, there is no significant difference in the length of stay between TRA and TFA.

A cross-national survey was undertaken among interventional radiologists in Europe and the United States to evaluate the utilization of RA within this community, along with its perceived benefits and drawbacks [33]. The survey demonstrated that the main barriers for TRA adoption by IRs are the perception that it is associated with longer learning curve, potential increased risk of stroke, longer procedural time and increase in radiation exposure. However, current literature indicates that these are only misperceptions and perceived limitations or obstacles [1–6, 33].

To encourage interventional radiologists to advocate for radial access, we suggest incorporating it into the IR training curriculum. IR trainees should spend part of their training in centers where radial access is frequently used. If this opportunity is not available, their training programs should collaborate with cardiology services to allow IR trainees to spend some time in the cath lab learning radial access.

We acknowledge that the relatively small sample size and lack of long-term follow-up data, particularly concerning complications such as radial artery occlusion, are limitations of our study. We are unable to extrapolate any of our data to female patients due to the nature of the procedure studied herein. This is a limitation of this study given that RAO risk is greater in smaller vessels and is associated with female sex. Equally we have no expertise nor experience in paediatric populations.

## Conclusion

In this article, we emphasize the importance of interventional radiologists optimizing the TRA to suit their interventional procedures. We recommend the use of sublingual GTN due to its efficacy in significantly dilating the radial artery, thereby facilitating puncture and reducing post-puncture spasm. Furthermore, we advocate for the use of ultrasound instead of the Babu test for quick and reliable assessment of arterial anatomy in the wrist and forearm. Compared to existing protocols learned from cardiologists, sublingual GTN is more practical and less time-consuming, with fewer side effects. These measures streamline the radial artery access procedure, making it quicker and more efficient for interventional radiologists.

## Data Availability

The datasets used and analysed during the current study are available from the corresponding author on reasonable request.

## Abbreviations

(T)FA: (Trans) femoral access
AVF: Arteriovenous fistula
CAGB: Coronary artery bypass graft
CFA: Common femoral artery
CVA: Cerebrovascular accident
DAP: Dose area product
DRA: Distal radial access
EMLA: Eutectic mixture of local anaesthesia
GTN: Glyceryl trinitrate
IPSS: International Prostate Symptom Score
IR: Interventional radiology
PAE: Prostatic artery embolisation
PCI: Primary coronary intervention
PGD: Patient group directive
QoL: Quality of Life
RA: Radial access
RAD: Radial artery diameter (mm)
RAO: Radial artery occlusion
TRA: Trans radial access
UFE: Uterine fibroid embolisation
